# POSA: Perl Objects for DNA Sequencing Data Analysis

**DOI:** 10.1186/1471-2164-5-60

**Published:** 2004-08-27

**Authors:** Jan A Aerts, Bart J Jungerius, Martien AM Groenen

**Affiliations:** 1 Animal Breeding and Genetics Group, Wageningen University, PO Box 338, 6700AH Wageningen, Netherlands; 2 Complex Genetics Group, Department of Biomedical Genetics, University Medical Centre, PO Box 80030, 3508 TA Utrecht, Netherlands

## Abstract

**Background:**

Capillary DNA sequencing machines allow the generation of vast amounts of data with little hands-on time. With this expansion of data generation, there is a growing need for automated data processing. Most available software solutions, however, still require user intervention or provide modules that need advanced informatics skills to allow implementation in pipelines.

**Results:**

Here we present POSA, a pair of new perl objects that describe DNA sequence traces and *Phrap *contig assemblies in detail. Methods included in POSA include basecalling with quality scores (by *Phred*), contig assembly (by *Phrap*), generation of primer3 input and automated SNP annotation (by *PolyPhred*). Although easily implemented by users with only limited programming experience, these objects considerabily reduce hands-on analysis time compared to using the Staden package for extracting sequence information from raw sequencing files and for SNP discovery.

**Conclusions:**

The POSA objects allow a flexible and easy design, implementation and usage of perl-based pipelines to handle and analyze DNA sequencing data, while requiring only minor programming skills.

## Background

Today, many genetics laboratories have access to modern capillary DNA sequencing machines, such as the ABI PRISM 3100, 3700 or 3730. These machines generate vast amounts of raw sequence data with little user intervention. Consequently, the amount of data to be analyzed has expanded and the bottleneck now is the analysis capacity. Data analysis capacity can be increased by higher levels of automation. Investments in infrastructures to process the raw sequencing data in sophisticated but rigid pipelines might be justified for larger laboratories and larger projects but might be too costly for smaller laboratories. In addition, rigid pipelines are too impractical if different projects share run-time on the same machine while requiring (slightly) different analysis procedures (e.g. vector trimming is needed in plasmid sequencing, but needless when sequencing PCR products).

Nucleotide sequence analysis can be performed with a variety of software tools. Although the number of console and web-based software tools has grown rapidly, the routine use of data input, output and storage may be inconvenient. Furthermore, for performing a series of analyses with different software tools, the sequence data need to be reformatted to the required data structure. Alternatively, sophisticated software suites that provide an integrated environment often are expensive.

Several of the available software solutions are designed to facilitate automated DNA sequence analysis at low cost. Well-known solutions are the Staden package and Bioperl.

The Staden Package contains *pregap4 *and *gap4*, full-featured applications with an intuitive graphical user interface [1]. These programs handle a list of raw sequence reads method-by-method. The programs in the Staden Package typically require a degree of user intervention and thus hands-on time.

Alternatively, Bioperl is a group of perl modules describing many genetics and genomics concepts [2]. For example, it includes the Bio::Seq::SeqWithQuality object that provides some of the basic properties of a raw sequence (i.e. its nucleotide sequence and quality values); the Bio::Tools::Primer3 object provides methods to work with primer3 input and output. However, to build custom DNA sequencing data pipelines, basic programming skills are needed to combine all these modules.

Smaller laboratory sites, however, often need to implement versatile pipelines that can be adjusted for any research question that suits the project best; at the same time, they often also do not have dedicated programmers available.

Although (semi-)automated procedures have been published by other groups [3,4], these are mostly focused on one particular pipeline and environment.

Here, we present POSA, a set of two new perl objects (Read.pm and Contig.pm) that describe a raw sequence and a *Phrap *contig in detail and are easily implemented in perl-based pipelines. Because these objects provide building blocks for sequencing data analysis pipelines and the actual pipelines are built using perl-scripts, the POSA objects can be used in very diverse settings.

## Implementation

The POSA source code is entirely coded in object-oriented Perl and consists of two objects: Read.pm and Contig.pm. In general, there are two important concepts associated with objects: methods (built-in procedures that can be performed on the object) and properties (describing some of the characteristics of the object). Most methods in the objects rely on the availability of other third-party programs (see Dependencies). Basically, POSA provides a wrapper around these programs and provides easy design and implementation of these programs in automated data analysis. The Read.pm object describes a DNA sequence trace and includes methods for data import from a variety of formats. It relies on *Phred *[5,6] for import and interpretation of raw sequence data. The original trace data are stored in binary (*scf*) format within the object. Other methods of Read.pm use modules of the Staden Package [1], such as *qclip *and *vector_clip *(if installed). Properties of Read.pm include e.g. the DNA sequence, quality scores, template and vector names and read direction.

The Contig.pm object contains a method to assemble contigs of reads using the *Phrap *program [6]. The object typically is created based on a list of Read.pm objects and can be exported as alignments or screened for polymorphisms using *PolyPhred *[7].

**Figure 1:**
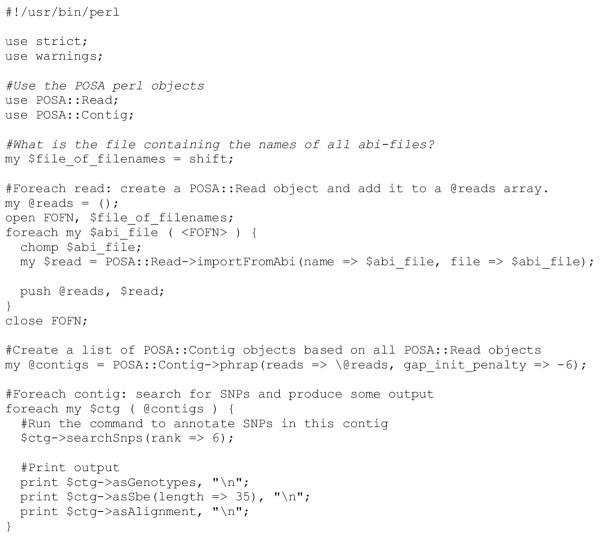
A typical script that takes a list of ab1 files for analysis and assembly, reports the contig, and lists the putative SNP positions and SBE primers.

**Figure 2:**
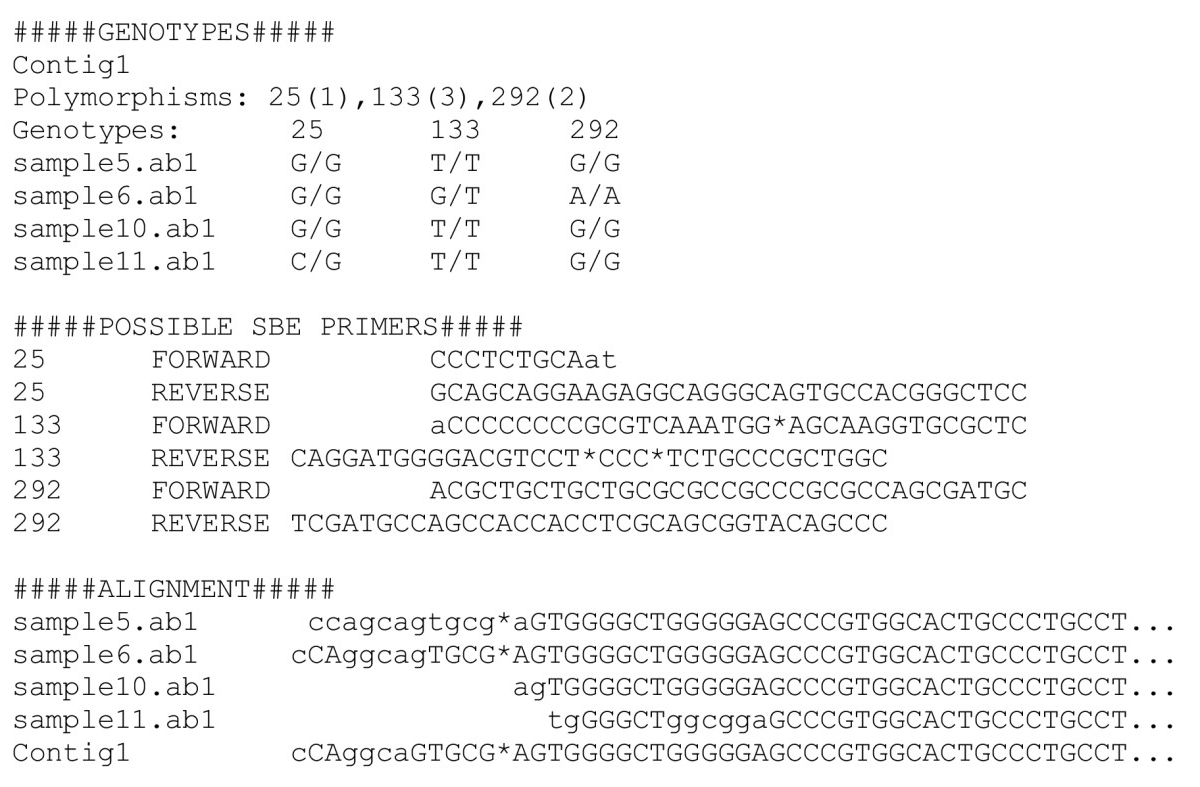
Typical output as generated by the script in Figure 1.

Both the Read.pm and Contig.pm objects were designed with flexibility in mind. To allow a (virtually) unlimited amount of data to be processed, the perl scripts using these objects work sequence-by-sequence rather than method-by-method. Typically, these objects are called from straightforward perl scripts that outline the analysis steps to be performed. Example scripts using the objects can be accessed from the download website. An example of a script and output using the two objects to process a set of reads and annotate sequence polymorphisms from the assembled contig is given in Figure 1 and Figure 2.

POSA was developed with perl 5.6.1 and tested on a SuSE linux 8.1 system for *abi*-files from the ABI PRISM 377 DNA Sequencer and 3100 Genetic Analyzer (Applied Biosystems). *Phred*, *Phrap *and *PolyPhred *versions were 0.000925.c, 0.990329 and 4.05, respectively.

## Results and Discussion

### Functionality

POSA provides an interface to design and implement automated sequencing data analysis. Sequencing data may be used in a variety of formats and originate from a variety of sources, e.g. data in *fasta, abi/ab1 *or *scf *format retrieved from websites or from newly generated traces. In addition, new objects can be initiated from a text file or can be opened from previous stored objects. Subsequently, a variety of methods can be applied, including basecalling and assessment of quality codes (by *Phred*), quality clipping, vector clipping, screening for *E. coli *(or other) sequence, contig assembly (by *Phrap*) and analysis. The method *asPrimer3 *can automatically generate input for the primer3 program [8] and is available in both objects. To facilitate automated SNP discovery or typing, the *SearchSnp*s method will generate output as shown in Figure 2. This method is based on the *PolyPhred *program and uses the 'rank' argument to set the stringency.

Finally, data can be stored in objects, or in files in either *exp*, *scf *or *fasta *format. In addition, the data can be saved in a *primer3 *input file to allow automated PCR primer design, or data can be saved in MIPE format (i.e. an XML format to store information on PCR experiments; see ). Data on assembled contigs can be exported as a list of reads in a contig, as consensus sequence, as alignment, as putative SNPs, as SBE primers for SNP genotyping or as *gff *file for visualization in Gbrowse [9]. Combinations of the diversity of input, analysis and output options allow for a wide spectrum of possible implementations. Examples of possible analysis pipelines include (but are not limited to) BAC-end sequencing with automated PCR primer design for chromosome walking and resequencing of PCR products with SNP annotation either for SNP genotyping or for SNP discovery and SBE primer design. Examples of scripts are provided on the web site .

### Performance

Although it represents only one of the numerous possible POSA-based pipelines, performance of POSA was validated by comparison of SNP discovery with the data after analysis using the Staden package. To do so, 5 PCR products were resequenced from a panel of 16 individuals to identify SNPs. Manual editing using the Staden Package revealed a total of 48 SNPs. Automated analysis using POSA also yielded a total of 48 SNPs with SNP ranking codes 1-3. Together, 41 SNPs were assigned with both manual editing and POSA. The remaining 7 SNPs assigned in manual editing corresponded to SNPs with ranks 4-6 in the POSA analysis. The 7 SNPs that were only assigned by POSA all originated from regions with lower quality sequence. While analysis time was reduced from several hours to a few minutes, POSA assigned SNPs in a way that was highly consistent with manual editing. This was expected because POSA provides options for an integrated analysis pipeline, but essentially is a wrapper around well-established sequence analysis tools like *Phred*, *Phrap *and *PolyPhred*.

### Intended use and benefits for users

POSA is a tool that provides easy and highly automated DNA sequence and contig data analysis using popular analysis tools. Automated sequence analysis reduces analysis time from several hours to a few minutes. Pipelines can easily be expanded or adapted through perl scripts. Writing or altering the perl scripts is straightforward to do for people with only basic computer skills, although more linux/unix experience might be necessary to install the required software (e.g. *Phred *and *Phrap*). Overall, this guaranties easy implementation of highly automated quality pipelines in combination with high flexibility in setup and design.

The perl objects are released under an open source license, allowing code improvements by the user community.

## Conclusions

POSA describes a DNA sequence read and a *Phrap *contig assembly in detail. These objects allow a flexible and easy setup of perl-based pipelines to handle DNA sequencing data, including generating primer3 input and automated SNP discovery, while requiring only little programming skills.

## Availability and requirements

Project name: POSA

Project home page: 

Operating system: platform independent Programming language: Perl 5.6.1

License: Artistic License (Open Source)

### Requirements

• Perl modules: Carp; Statistics::Descriptive; Tie::File; IO::File; POSIX:: Storable.

• Phred, Phrap, PolyPhred

• Pregap4, gap4 (Staden Package (optional))

• Primer3 (optional)

## List of abbreviations

POSA Perl objects for DNA sequencing data analysis

SNP single nucleotide polymorphism

abi/ab1ABI PRISM trace file format

scf standard chromatogram format

exp experiment file format, developed by Staden (see )

MIPE minimum information on PCR experiments (see )

BAC bacterial artificial chromosome

PCR polymerase chain reaction

SBE single base extension

## Authors' contributions

JA programmed the Perl objects and participated in development of concept and architecture of the software; BJ participated in development of concept and architecture and wrote the manuscript; MG supervised the project. All authors read and approved the final manuscript.
